# Influence of Mg Doping on the Structure and Mechanical Properties of Al_2_Cu Precipitated Phase by First-Principles Calculations

**DOI:** 10.3390/ma17010093

**Published:** 2023-12-23

**Authors:** Jiyi Li, Yan Huang, Xuan Zhang, Liang Zhang

**Affiliations:** 1 International Joint Laboratory for Light Alloys (MOE), College of Materials Science and Engineering, Chongqing University, Chongqing 400044, China; 2 Shenyang National Laboratory for Materials Science, Chongqing University, Chongqing 400044, China

**Keywords:** first-principles calculations, structural stability, mechanical property, thermodynamic

## Abstract

Al-Cu-Mg high-strength alloys are widely used in industrial production because of their excellent mechanical performance and good machining properties. In this study, first-principles calculations based on density functional theory were carried out to investigate the influence of Mg doping on the structural stability and mechanical properties of the Al_2_Cu (θ) precipitated phase in Al-Cu-Mg alloys. The results show that the structural stability, electronic structure, bulk modulus, mechanical anisotropy, and thermodynamic properties of the precipitated Al_2_CuMg_X_ phase change with the concentration of Mg doping (X = 2, 4, 6, and 8). The cohesive energy calculation and electronic structure analysis show that Al_2_CuMg_6_ has a high structural stability. The criterion based on elastic constants indicates that Al_2_CuMg_2_, Al_2_CuMg_4_, and Al_2_CuMg_8_ have a brittle tendency and show strong anisotropy of mechanical properties, while Al_2_CuMg_6_ shows better comprehensive mechanical properties. The thermodynamic analysis results based on the quasi-harmonic Debye model show that the Al_2_CuMg_6_ precipitated phase has good stability at high temperatures and pressure.

## 1. Introduction

As a kind of high-performance material, Al-Cu-Mg high-strength aluminum alloy has been widely used in aerospace, weapon equipment, new energy vehicles, and other fields due to its excellent mechanical properties and good machinability. After the supersaturated solid solution is formed by solution treatment or quenching, the supersaturated solute atoms are diffused and precipitated in the aging process and form the precipitated phase. For Al-Cu-Mg alloys, different types of strengthening phases can be precipitated in the aging process according to the alloy components, such as θ and θ′ phases (Al_2_Cu), θ″ phase (Al_3_Cu_2_), S phases (Al_2_CuMg), and Ω phases (Al_2_CuMgAg) [1,2,3,4,5]. These precipitated phases have an important effect on the properties of the Al-Cu-Mg alloy.

Previous researchers have studied the evolution of doping and precipitated phases in Al-Cu-Mg alloys. For example, Cai et al. [6] investigated the effect of Ag on the precipitation behavior of the Ω phase in Al-Cu-Mg alloys. It was found that Ag could promote the precipitation of the Ω phase and increase the activation energy of the θ′ phase, thus inhibiting the precipitation of the θ′ phase. Xiao et al. [7] analyzed the effect of different concentrations of Cu and Mg doping on the aging behavior of Al-Cu-Mg alloys. The results indicate that different types of precipitated phases can be obtained by adjusting the Cu and Mg concentrations. Zhu et al. [8] investigated the formation process of the S precipitated phase in Al-Cu-Mg alloys using the first-principles method. The results show that substituting Zr atoms for Al atoms on the (021) surface can promote the precipitation of the S phase and enhance its bulk modulus and shear modulus. Therefore, elemental doping has an important effect on the structural stability and mechanical properties of Al-Cu-Mg alloys.

Al_2_Cu (θ phase) is one of the common precipitated phases in Al-Cu-Mg alloys [9,10], and its structure and properties were first described by Friaul et al. [11]. They found that after solid solution treatment, the θ phase is desaturated and precipitated from the matrix during aging to form clusters of solute atoms, leading to the hardening of the alloy. Liu et al. [12] showed that Al_2_Cu could effectively improve the corrosion resistance of materials. It is also beneficial for the recrystallization of the microstructures and improves the fracture toughness of the material. Chen et al. [13] showed that doping elements would affect the nucleation, stability, and properties of the θ phase. They found that both co- and semi-co-grid interfaces can be formed between Al_2_Cu, and solute elements show different bias behavior at the Al_2_Cu interface. In addition, computational simulations were carried out to investigate the properties of the Al_2_Cu precipitated phase. Perakis et al. [14] investigated the interfacial structures of the θ phase in Al-Cu alloys at high temperatures after long aging using transmission electron microscopy and first-principles calculations. It was found that the lateral growth of the θ phase is influenced by several major mechanisms. Atomic diffusion of the θ phase transition can occur either through a gap mechanism based on additional Al atoms or directly through Al atom diffusion, both of which are quite different from previous theories based on direct diffusion of Cu atoms. Shao et al. [15] studied the effect of Nb, Ge, and Ni doping on the structural, stability, mechanical, and electronic properties of the Al_2_Cu precipitated phase by using first-principles calculations. The results show that doped elements improve the stiffness of the precipitated phase but reduce its ductility. In addition, doping elements can greatly reduce the anisotropy of the precipitated phase and significantly improve its binding strength and stability.

Due to the constraints of the variety of precipitated phases, complex structure, and difficulty of characterization, it is not easy to fully understand the relationship between the structure and properties of precipitated phases through experiments and characterization methods, which restricts the further development of high-performance alloys. The atomistic simulation method, such as first-principles calculations, provides a new idea for the composition design of alloys. The calculation method has been proven to be effective in studying the influence of doping elements on the precipitated phase [16,17,18,19] and can be used to optimize the structure and study the properties [20]. In this paper, the effect of Mg doping on the structural stability, bulk modulus, mechanical properties, and electronic structure of the Al_2_Cu (θ phase) have been studied by first-principles calculations, which provides theoretical guidance for composition design and property optimization of the high-performance Al-Cu-Mg alloy.

## 2. Materials and Methods

First-principles calculations were performed using the projector-augmented plane wave (PAW) method in the Vienna ab initio simulation package (VASP) code [21,22]. The exchange-correlation term is treated with the Perdew–Burke–Ernzerhof (PBE) function [23]. At this point, the cutoff energy of the plane wave is set to 700 eV to ensure the accuracy of the results. The supercell of 2 × 2 × 2 (Al_64_Cu_32_) was built to achieve low dopant element concentration. The integration in the inverse space was performed using a Monkhorst–Pack grid centered at the Γ-point with a K-spacing of 0.1 Å^−1^. The convergence criterion for the total energy is 10^−8^ eV. All the related structures have been relaxed using the conjugate gradient algorithm. The structural optimization was performed until all residual forces acting on each atom’s residual forces are less than 1 meV/Å. The electronic configurations of the potentials utilized in the analysis are Al (*3s*^2^ *3p*^1^), Cu (*3d*^10^ *4s*^1^), and Mg (*2s*^2^ *2p*^6^).

**Figure 1:**
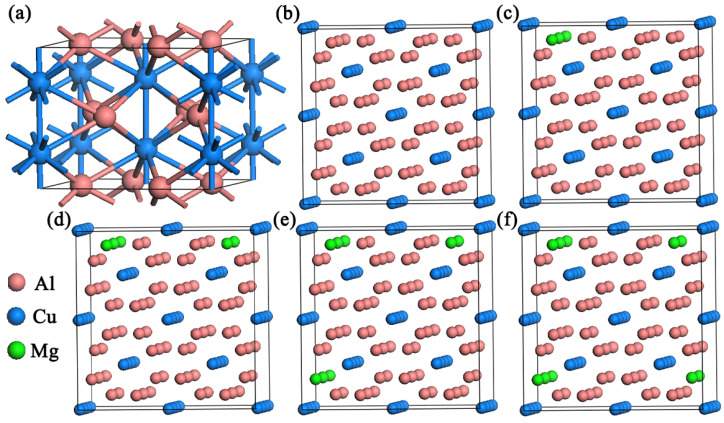
(**a**) The crystal structure of the Al_2_Cu precipitated phase. (**b**) The crystal structure of supercell structure Al_2_Cu 2 × 2 × 2 supercell. Configuration of the doped phase: (**c**) Al_2_CuMg_2_. (**d**) Al_2_CuMg_4_. (**e**) Al_2_CuMg_6_. (**f**) Al_2_CuMg_8_.

The experimentally determined θ phase has a tetragonal structure with space group I4/mcm (No. 140) and t/12 symmetry. Its unit cell contains 4 formula units, as shown in Figure 1a. The lattice constants are a = 6.063 Å and c = 4.872 Å. Al and Cu atoms are located on θ (0.1541, 0.6541, 0) and 4a (0, 0, 0.25) sites, respectively, and Figure 1b is the supercell model. The lattice constants obtained after the crystal structure optimization are shown in Table 1, which agrees well with the previous experimental results [24]. In order to study the preferential site occupation of Mg addition in the Al_2_Cu phase, the formation energies of the impurities-doped Al_2_Cu phases were calculated through ${\Delta H=E}_{total}^{AB}-[x_{A}E_{solid}^{A}+(1-x_{A})E_{solid}^{B}]$ [25]. Lower formation energy corresponds to a more preferential site occupation tendency. When the doping Mg atom occupied the Al and Cu site, the formation energy was 4.43 eV and 8.62 eV per atom, respectively. It indicates that the doping Mg atom prefers to occupy the Al site in the Al_2_Cu structure. The change in Mg concentration in the Al_2_Cu precipitated phase is realized by changing the number of Mg atoms doped in the model. In this study, the number of Mg atoms doped in Al_2_CuMg_X_ is set as X = 2, 4, 6, 8. The different concentration models are shown in Figure 1c–f, where two Mg atoms are defined as a set to obtain different doping concentrations from 2 to 8 Mg atoms, and the doped Mg atoms are distributed discretely between each other.

## 3. Results and Discussion

### 3.1. Structural Properties

The structural stability of the precipitated phase is closely associated with its cohesive energy (E_coh_), which is the energy released during the process of atoms combining from a free state into a crystalline compound [28]. The negative value of cohesive energy indicates that the structure can exist stably, thus indicating the structural stability of the Al_2_Cu precipitated phase in this study. The larger the absolute value of cohesive energy, the more stable the structure. E_coh_ can be obtained by Equation (1).

(1) $$E_{coh}=E_{total}^{AB}-[x_{A}E_{atom}^{A}+(1-x_{A})E_{atom}^{B}]$$

where E_coh_ is the cohesive energy, $E_{total}^{AB}$ is the energy per number of atoms for each intermetallic compound, $E_{atom}^{A}$ and $E_{atom}^{B}$ are the energy of free atoms A and B, and X_A_ represents the atomic fraction of A in compounds. From the calculation results shown in Table 1, it can be seen that the cohesive energy of the Al_2_Cu phase is −3.82 eV/atom, which agrees with the previous calculation results [27]. In addition, the value of cohesive energy of Al_2_Cu doped with different Mg concentrations is negative and lower than that of the Al_2_Cu phase, which means that the doped Al_2_Cu phases can exist stably, and Mg can improve the stability of the Al_2_Cu phase. In addition, the absolute values of E_coh_ of Al_2_CuMg_6_ and Al_2_CuMg_8_ are greater than that of Al_2_CuMg_2_ and Al_2_CuMg_4_, indicating the configurations of Al_2_CuMg_6_ and Al_2_CuMg_8_ are more stable than the other two configurations.

**Figure 2:**
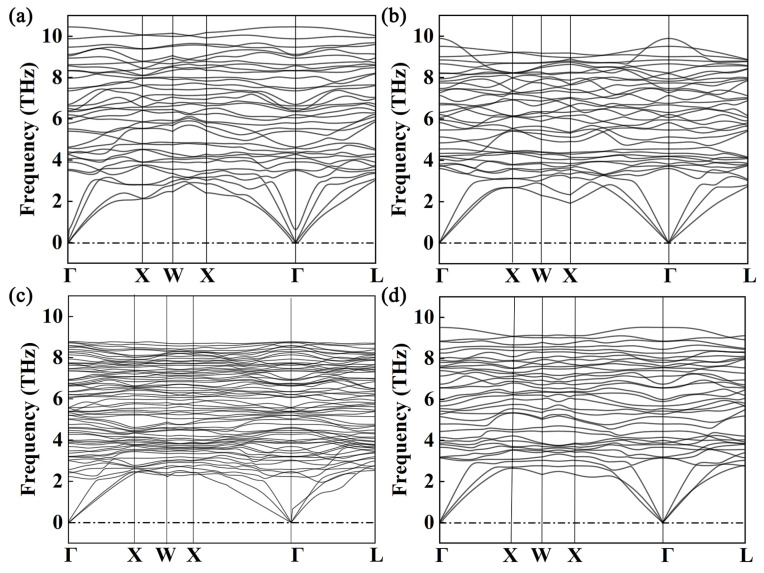
Phonon spectrum calculation of (**a**) Al_2_CuMg_2_, (**b**) Al_2_CuMg_4_, (**c**) Al_2_CuMg_6_, and (**d**) Al_2_CuMg_8_.

In order to investigate the dynamical stability of the crystal of Al_2_CuMg_X_, the phonon spectra are calculated by first-principles calculations. The model established in this work is a doping model, and the symmetry corresponding to different concentration gradients is not the same, and the symmetry is related to the acoustic branch and the optical branch of the phonon spectrum. The abscissa of the phonon spectrum is taken as the point of high symmetry, and the ordinate is the frequency of the phonon spectrum. The acoustic branch represents the overall vibration of the primitive cell, while the optical branch represents the relative vibration between atoms within the primitive cell. Based on the determined lattice constants, the phonon dispersion for the 2 × 2 × 2 supercell of Al_2_CuMg_X_ is shown in Figure 2. Note that the phonon-related properties in this work are calculated based on the density functional perturbation theory. Figure 2 shows that no negative vibrational modes appear in the phonon spectrum, and there are no imaginary frequencies, indicating the dynamical stability of the cubic Al_2_CuMg_X_.

### 3.2. Electronic Structure

**Figure 3:**
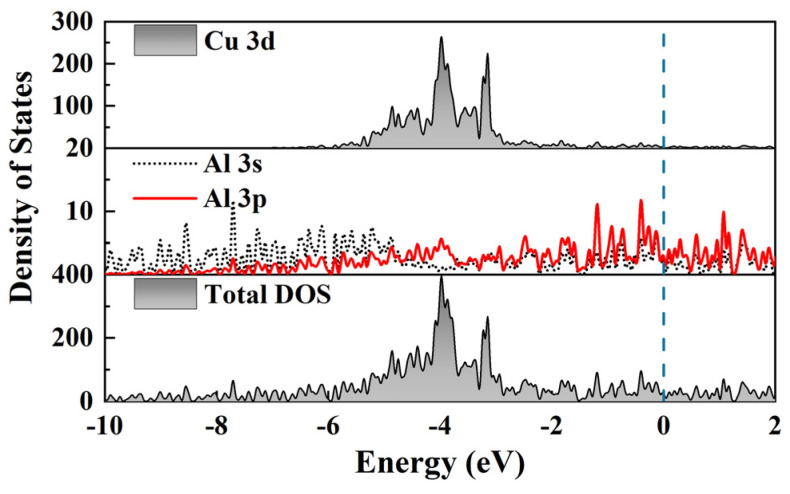
Total and partial density of state of Al_2_Cu. The dashed line is the Fermi level.

**Figure 4:**
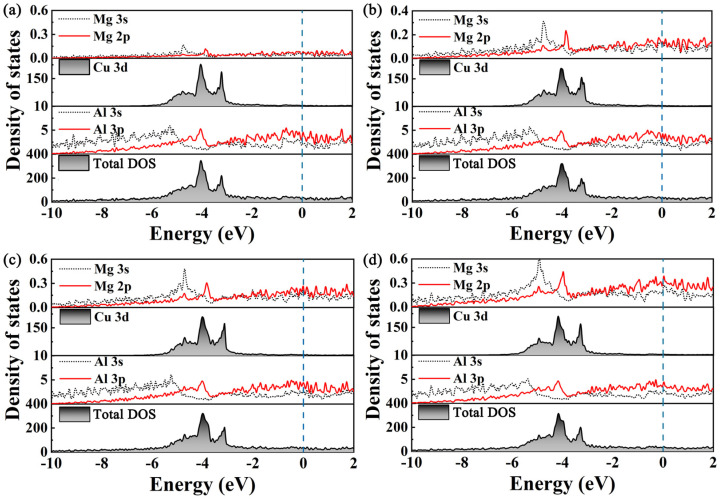
Total and partial density of state of Al_2_CuMg_X_: (**a**) Al_2_CuMg_2_, (**b**) Al_2_CuMg_4_, (**c**) Al_2_CuMg_6_, and (**d**) Al_2_CuMg_8_. The dashed line is the Fermi level.

In order to reveal the bonding characteristics of Al_2_CuMg_X_ and further understand the structural stability differences of the three phases, their partial density of states (PDOS) and total density of states (DOS) were calculated, respectively, as shown in Figure 3 and Figure 4. As shown in Figure 3, there is a peak in the total density of states, located in the range of −6 to −2 eV, which is mainly caused by the *3d* orbitals of Cu, and the hybridization between electrons is not very obvious. The free electrons at the Fermi level mainly come from the *p*-orbitals of Al *3p*.

The spin-resolved total and density of states (DOS) of the Mg-doped Al_2_Cu phases were calculated by the tetrahedron method with Blöchl corrections. The DOS for all the cases were calculated by their equilibrium lattice constants and optimized internal parameters. The Fermi level (E_f_) was set as zero and was used as the reference. The valence electrons of the orbital electrons contributing to the calculations are Al (*3s*^2^ *3p*^1^), Cu (*3d*^10^ *4p*^1^), and Mg (*2s*^2^ *2p*^6^).

In addition, structural stability is closely related to the electrons’ orbital contribution. To further interpret the contributions of different orbitals to the DOS of the Al_2_Cu precipitated phases, the PDOS at atomic resolution is presented in Figure 4. The common feature of the four configurations is that there are several characteristic peaks in the DOS. The width of the peak is mainly between −6 and −2 eV, and the peak is relatively narrow. The peak values are 403.26, 372.65, 364.42, and 387.54 states/eV, respectively. This is a typical characteristic of transition metals caused by the *d* electron orbits of Cu, indicating that the *d* electrons are relatively localized. The binding peaks between −6 and −2 eV principally originate from the contribution of Al (*3s* and *3p*) and Mg (*2s* and *2p*) orbits. The hybridization between the electrons in all precipitated phases is not apparent. The free electrons near the Fermi level mainly come from the contributions of the state electrons of Al (*3p*) and Mg (*2p*), and as the Mg concentration increases, more free electrons are generated, leading to a certain degree of hybridization and increasing the stability of the precipitate phase, which is consistent with the results of cohesive energy.

### 3.3. Elastic Properties

Elastic constants (C_ij_) are investigated to understand the mechanical and physical properties of the Al_2_Cu precipitated phase with doping Mg atoms. Elastic constants reflect the bond strength between atoms and are dependent on the effect of external forces on the crystal, playing an important role in determining the mechanical properties of materials. There is a quadratic linear relationship between energy and lattice strain in the crystal structure. Based on the energy-strain relationship, a certain elastic constant or a combination of crystal elastic constants can be obtained by applying strains in different directions and sizes to crystals, and finally obtaining the constant flexible matrix of the crystal. Some criteria are utilized to judge the mechanical stability of the precipitated phases based on the elastic constants. For tetragonal structures, the criteria are determined by Equation (2) [29]:

(2) $$C_{11}>0,\;C_{33}>0,\;C_{44}>0,\;C_{66}>0,\;\left(C_{11}-C_{12}\right)>0,\;\left(C_{11}{+C}_{33}-{2C}_{13}\right)>0,\;\left[2\left(C_{11}+C_{12}\right)+C_{33}+4C_{13}\right]>0$$

The calculated elastic constants of Al_2_CuMg_X_ are listed in Table 2. According to the elastic constants and mechanical stability criteria, all of them satisfy Born’s criterion [30], indicating that they can exist stably, which is consistent with the calculation results of cohesive energy.

The elastic properties of the precipitated phases were obtained based on the calculations of the elastic constants of the precipitated phases and using the Voigt–Reuss–Hill approximation [31], as shown in Table 3. For the tetragonal crystal system, the bulk modulus (B) and the shear modulus (G) can be calculated by Equations (3)–(10) [32]:

(3) $$M=C_{11}+C_{12}+2C_{33}-4C_{13}$$

(4) $$C^{2}=\left(C_{11}+C_{12}\right)C_{13}-2C_{13}^{2}$$

(5) $$B_{V}=\left(2C_{11}+2C_{12}+C_{33}+4C_{13}\right)/9$$

(6) $$G_{V}=\left(M+3C_{11}-3C_{12}+12C_{44}+6C_{66}\right)/30$$

(7) $$B_{R}=C^{2}/M$$

(8) $$G_{R}=15{\left(\frac{18B_{v}}{C^{2}}+\frac{6}{C_{11}-C_{12}}+\frac{6}{C_{44}}+\frac{3}{C_{66}}\right)}^{-1}$$

(9) $$B=\left(B_{V}+B_{R}\right)/2$$

(10) $$G=\left(G_{V}+G_{R}\right)/2$$

The theoretical value of the modulus of elasticity can be approximated by Equations (11) and (12) [33]:

(11) $$B_{H}=\frac{B_{V}+B_{R}}{2}$$

(12) $$G_{H}=\frac{G_{V}+G_{R}}{2}$$

Poisson’s ratio (ν) and elastic modulus (E) can be calculated according to Equations (13) and (14) [33]:

(13) $$\nu =\frac{{3B}_{H}-2G_{H}}{2({3B}_{H}+G_{H})}$$

(14) $$E=\frac{{9B}_{H}G_{H}}{{3(B}_{H}+G_{H})}$$

High bulk modulus (B) indicates that the material has strong resistance to volume change, while shear modulus (G) represents the crystal’s resistance to plastic deformation, both of which are closely related to the hardness of materials. The high values of B and G reflect the strong bond between atoms. In Table 3, it can be seen that with the addition of the Mg element, the bulk modulus, shear modulus, and Young’s modulus of Al_2_Cu are all improved. The values of the bulk modulus and shear modulus of Al_2_CuMg_8_ are 58.87 and 60.42 GPa, and the values of Al_2_CuMg_6_ are 60.42 and 37.59 GPa, respectively, which are higher than the values of the other two configurations of precipitated phases. The results indicate that Al_2_CuMg_6_ and Al_2_CuMg_8_ could show good compressive and shear resistance properties. In addition, the hardness of the material (Hv) is closely related to the elasticity of the material, which can be predicted by a semi-empirical formula Hv = 2(K^2^·G_H_)^0.585^ − 3.0, where K = G_H_/B_H_ and G_H_ is the shear modulus [34]. The hardness values of Al_2_CuMg_X_ with different Mg concentrations are 20.35, 21.69, 23.58, and 24.09 GPa, respectively.

According to Pugh’s theory, the ductility of the materials is closely related to the B/G value, where a value higher than 1.75 shows a favorable ductility [35]. The results displayed in Table 3 reveal that with the increase in Mg concentration, a gradual transition to a ductile phase becomes apparent. However, only Al_2_CuMg_6_ is the ductile phase, and Al_2_CuMg_2_, Al_2_CuMg_4_, and Al_2_CuMg_8_ are the brittle phases, which indirectly indicates that concentration changes affect the mechanical properties of the precipitated phase. Furthermore, the value of C_11_-C_12_ can also be used to determine the ductility of materials [36]. The smaller value means the better ductility of the materials. From Table 3, the value again proves that Al_2_CuMg_6_ has the best ductility. In addition, Poisson’s ratio (ν) is also an important index to measure the ductility or brittleness of materials. The high ductility is associated with a high value of Poisson’s ratio. According to the above criteria, it is believed that Al_2_CuMg_6_ is the most likely precipitated phase component to exhibit good ductility.

### 3.4. Elastic Anisotropy

The elastic anisotropy of materials is closely related to the initiation and propagation of micro-cracks in the process of deformation. The universal anisotropy index (A^U^) was introduced to describe the elastic anisotropy behavior of the Al_2_CuMg_X_ precipitated phases. A^U^ is calculated by Equations (15)–(17) [37]:

(15) $$A^{U}=5\frac{G_{V}}{G_{R}}+\frac{B_{V}}{B_{R}}-6$$

(16) $$A_{B}=\frac{B_{V}-B_{R}}{B_{V}+B_{R}}$$

(17) $$A_{G}=\frac{G_{V}-G_{R}}{G_{V}+G_{R}}$$

where B_V_ and B_R_ are the Voigt and Reuss bulk modulus, respectively. G_V_ and G_R_ are the Voigt and Reuss shear modulus [38]. Note that an isotropic material has A^U^ = 0, while the magnitude of any deviation from zero indicates the degree of elastic anisotropy. If the value of A^U^ is equal to zero, the system is isotropic. On the contrary, the more significant the difference from zero, the greater the anisotropy. It can be seen from Table 4 that the calculated A^U^ of Al_2_CuMg_6_ is 0.24, which is the lowest among the four configurations, implying the best elastic isotropy. The A^U^ value of Al_2_CuMg_8_ is the highest (0.45), indicating that the Al_2_CuMg_8_ exhibits strong anisotropy and is prone to generating micro-cracks.

**Figure 5:**
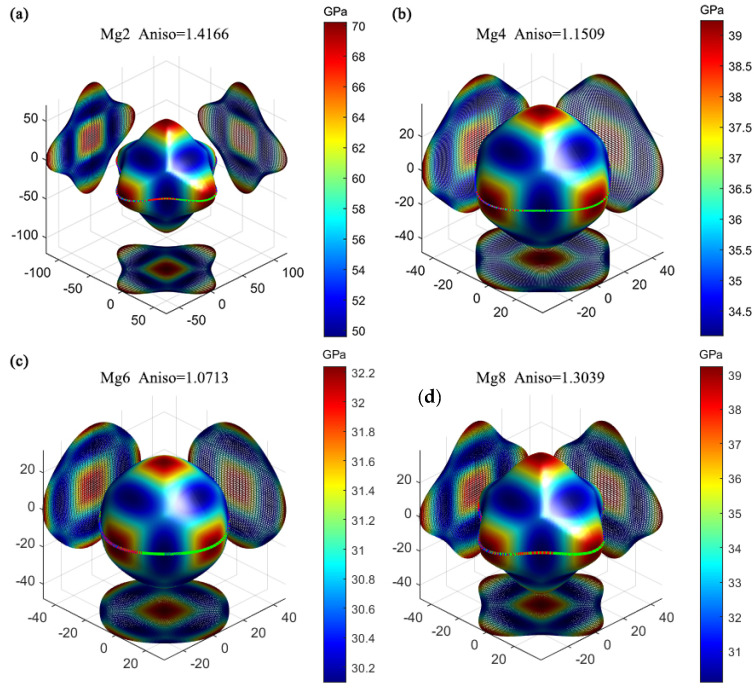
Three-dimensional diagram of Young’s modulus of the doped Al_2_Cu precipitated phase with different Mg concentrations: (**a**) Al_2_CuMg_2_, (**b**) Al_2_CuMg_4_, (**c**) Al_2_CuMg_6_, and (**d**) Al_2_CuMg_8_.

To further discuss the mechanical anisotropy of the Al_2_CuMg_X_ precipitated phases in different orientations, we used the spherical coordinate method to give the three-dimensional direction of Young’s modulus and the two-dimensional projections in the (001), (100), and (010) planes, as shown in Figure 5. The color bar represents the magnitudes of Young’s modulus. Based on the directional dependence of the three-dimensional closed surface on the elastic modulus, the degree of elastic anisotropy can be evaluated, and the relationship between S_ij_ and C_ij_ is an inverse matrix. The directional dependence of Young’s modulus for a cubic crystal can be obtained by Equations (18) and (19) [39]:

(18) $$E^{-1}={\left(1-l_{3}^{2}\right)}^{2}S_{11}+l_{3}^{4}S_{33}+l_{3}^{2}\left(1-l_{3}^{2}\right)\left(2S_{13}+S_{44}\right)$$

(19) $$E^{-1}=S_{11}-\left(2S_{11}-2S_{12}-S_{44}\right)\left(l_{1}^{2}l_{2}^{2}+l_{1}^{2}l_{3}^{2}+l_{2}^{2}l_{3}^{2}\right)$$

where S_ij_ are the elastic compliance constants and li (i = 1, 2, 3) are the direction cosines [39]:

l_1 =_
*sinθcosϕ* (20)

l_2 =_
*sinθsinϕ* (21)

l_3 =_
*cosθ* (22)

In an isotropic system, the 3D closed surface representing the relation between the elastic modulus and the crystallographic direction should be spherical. The degree of deviation from the sphere reflects the degree of anisotropy. Aniso represents the anisotropy of materials, and crystal anisotropy refers to the periodicity and density of the atomic arrangement along different directions of the lattice, resulting in different physical and chemical properties of crystals in different directions, and aniso uses value 1 as a reference. It can be combined and compared with A^U^ values to evaluate the anisotropy of Al_2_CuMg_X_. As shown in Figure 5, Young’s modulus of Al_2_CuMg_6_ deviates slightly from the sphere with the 3D characterization of crystallographic direction, and the value of aniso is 1.0713, which is the smallest among the four cases, indicating that the configuration has weak elastic anisotropy. As shown in Figure 5a,b,d, Al_2_CuMg_2_, Al_2_CuMg_4_, and Al_2_CuMg_8_ deviate noticeably from the spherical shape. The result indicates that these configurations have strong elastic anisotropy, which is negative to the ductility or toughness of the materials.

### 3.5. Thermodynamic Analysis

The above calculation results show that Al_2_CuMg_6_ has the best comprehensive mechanical properties among the four models of the Al_2_Cu precipitated phase. The thermodynamic properties of Al_2_CuMg_6_ were further investigated using the quasi-harmonic Debye model for Gibbs simulation [40,41]. The relationship between energy and volume, as well as Poisson’s ratio, were obtained through the matrix calculation of Al_2_CuMg_6_. The Brich–Murnaghan state equation was used for fitting to obtain the E-V curve [42]:

(23) $$E\left(V\right)=E_{0}+\frac{9V_{0}B_{0}}{16}\{[{\left(\frac{V_{0}}{V}\right)}^{\frac{2}{3}}-1{]}^{3}B_{0}^{\prime }+[{\left(\frac{V_{0}}{V}\right)}^{\frac{2}{3}}-1{]}^{2}\left[{6-4\left(\frac{V_{0}}{V}\right)}^{\frac{2}{3}}\right]\}.$$

**Figure 6:**
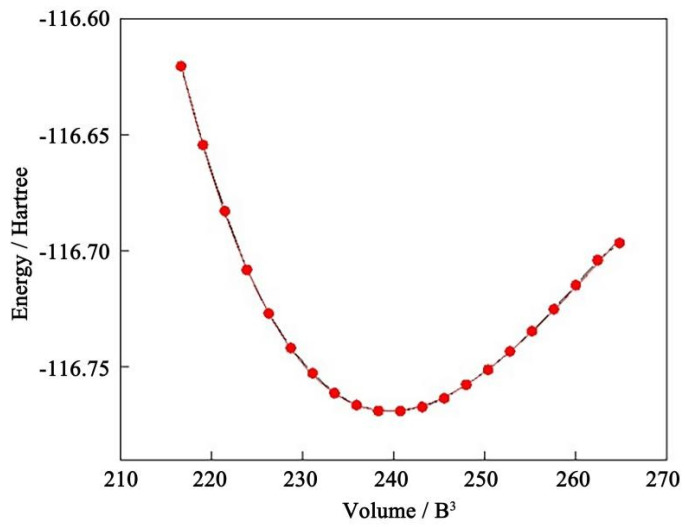
The relationship between energy and volume (E-V) curve of Al_2_CuMg_6_ phase.

The simulation temperature was set as 0 to 2000 K, and the system pressure was set as 0 to 20 GPa for the first-principles calculations. Although there are some errors in the results of the quasi-harmonic Debye model used in the thermodynamic calculations, they are in the same order of magnitude as the experimental values [43], so the fluctuations and trends of the thermodynamic properties of the precipitated phase can be predicted approximately. The E-V curve of the Al_2_CuMg_6_ precipitated phase was plotted in Figure 6. The lowest energy point was determined according to the fitting of the E-V curve. The results show that the fitting degree of the E-V curve is high, and the midpoint of volume is the lowest point of energy, indicating that the calculation method and parameters are accurate, which is the premise of thermodynamic analysis.

**Figure 7:**
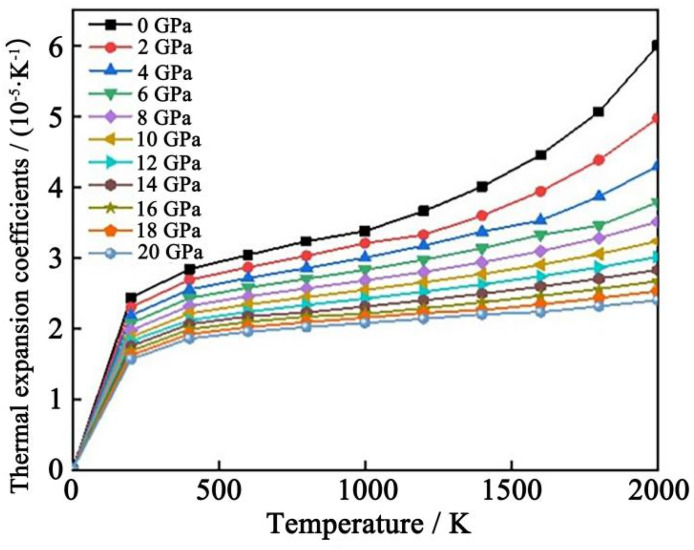
Thermal expansion coefficient of Al_2_CuMg_6_ phase as a function of temperature at different pressures.

Figure 7 shows the variation of the thermal expansion coefficient of Al_2_CuMg_6_ with temperature under different pressures. It is found that temperature and pressure have important effects on the thermal expansion coefficient of the precipitated phase structure. Before 500 K, the coefficient of thermal expansion changes rapidly with temperature. There is an obvious slope inflection point in the curve, and it basically occurs in the same temperature range. When the temperature rises above 500 K, the change rate of thermal expansion coefficient decreases continuously. When the pressure is at a relatively low level, the coefficient of thermal expansion is sensitive to the trend of temperature. However, when the pressure increases to more than 6 GPa, the thermal expansion coefficient tends to be flat with the increase in temperature, and the greater the pressure, the smaller the thermal expansion coefficient.

**Figure 8:**
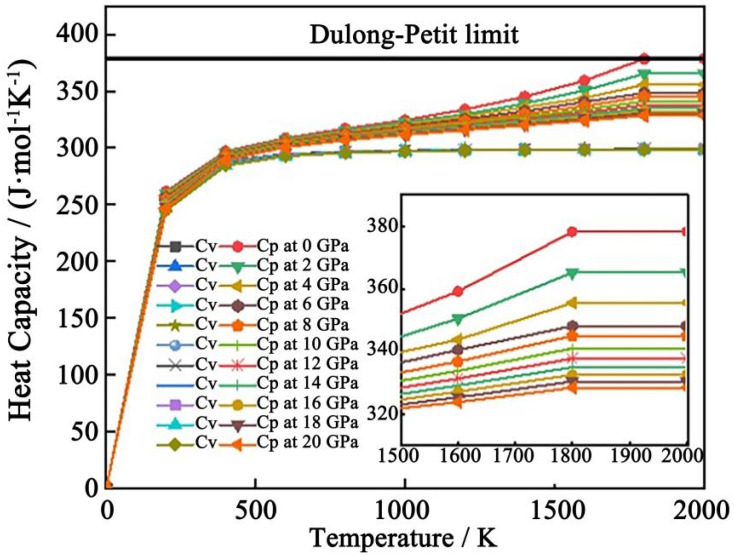
Heat capacity of Al_2_CuMg_6_ phase as a function of temperature at different pressures.

Figure 8 shows the isobaric heat capacity (C_p_) and isothermal heat capacity (C_v_) of Al_2_CuMg_6_ under different pressures as a function of temperature. As can be seen from the result, the values of C_v_ and C_p_ of the precipitated phase are basically the same in the low-temperature region below 300 K, and their values increase very fast due to the quantum effect at low temperatures. However, as the temperature continues to rise, the two curves show different trends, and the value of C_p_ increases continuously with the increase in temperature, while C_v_ gradually increases slowly, and finally tends to a saturation value. In the high-temperature region, the value of C_p_ is higher than C_v_, but the increasing speed also slows down with the temperature, which conforms to the Dulong–Petit law, indicating that the Al_2_CuMg_6_ precipitated phase has good stability at high temperature and high pressure.

## 4. Conclusions

First-principles calculations were carried out to investigate the structural stability, mechanical properties, and thermodynamic properties of the Al_2_Cu precipitated phase with different concentrations of Mg doping. The main conclusions are summarized as follows:

(1) The calculations of cohesive energy and phonons show that the Al_2_CuMg_X_ precipitated phase with different Mg doping can exist stably, and Al_2_CuMg_6_ shows the highest structural stability.
(2) The results of DOS analysis show that the changes in concentration have similar contributions to the electronic structure orbitals and hybridization effects of the precipitates, and the peaks are caused by Cu(*3d*); the electron hybridization of the precipitated phase is not obvious.
(3) Based on the Voigt–Reuss–Hill approximation criterion, the elastic constants of Al_2_CuMg_X_ were calculated. C_12_–C_44_, B/G, and other criteria show that Al_2_CuMg_6_ has better comprehensive mechanical properties compared to the other three precipitated phases. Mechanical anisotropy analysis shows that the anisotropy of Al_2_CuMg_6_ is the least significant among the four Mg doping precipitated configurations.
(4) The thermodynamic analysis results based on the quasi-harmonic Debye model show that the volume thermal expansion coefficient and heat capacity of Al_2_CuMg_6_ decrease with an increase in pressure and increase with an increase in temperature, which accords with the Dulong–Petit law, indicating that the Al_2_CuMg_6_ precipitated phase has good stability at high temperature and high pressure.

## Figures and Tables

**Table 1:** Lattice parameters and cohesive energy (E_coh_) of the doped Al_2_Cu phase.

Model	a (nm)	b (nm)	c/a	E_coh_ (eV/atom)
Al_2_Cu	5.9863	5.9863	0.7986	−3.82
	6.0630 [26]	6.0630 [26]	0.8036 [26]	−3.99 [27]
Al_2_CuMg_2_	6.0489	6.0489	0.8003	−3.95
Al_2_CuMg_4_	6.0495	6.0495	0.7998	−3.99
Al_2_CuMg_6_	6.0502	6.0502	0.7995	−4.09
Al_2_CuMg_8_	6.0512	6.0512	0.7992	−4.06

**Table 2:** The calculated elastic constants (C_ij_) of the precipitated phases (GPa).

Model	C_11_	C_12_	C_13_	C_22_	C_23_	C_33_
Al_2_Cu	144.22	82.24	56.24	132.68	59.09	209.75
Al_2_CuMg_2_	120.53	82.54	65.23	118.56	70.29	228.65
Al_2_CuMg_4_	119.65	91.23	71.2	129.95	61.02	237.32
Al_2_CuMg_6_	118.3	90.87	78.32	123.58	70.25	239.61
Al_2_CuMg_8_	126.9	91.26	65.46	119.85	63.24	229.65

**Table 3:** The calculated mechanical properties of Al_2_CuMg_X_.

Model	Modulus (GPa)			B/G	H_v_ (GPa)	C_11_–C_12_	Poisson Ratio
	B	G	E				
Al_2_Cu	53.82	33.95	81.77	1.63	19.54	61.98	0.30
Al_2_CuMg_2_	55.69	35.32	87.47	1.60	20.35	37.99	0.24
Al_2_CuMg_4_	57.14	35.09	87.37	1.69	21.69	28.42	0.25
Al_2_CuMg_6_	60.42	37.59	86.78	1.76	23.58	27.43	0.26
Al_2_CuMg_8_	58.87	39.58	87.30	1.66	24.09	35.64	0.24

**Table 4:** The calculated global anisotropy index (A^U^) and partial anisotropy index (A_G_ and A_B_).

Model	A^U^	A_B_	A_G_
Al_2_CuMg_2_	0.42	0.12	0.01
Al_2_CuMg_4_	0.35	0.17	0.06
Al_2_CuMg_6_	0.24	0.04	0.02
Al_2_CuMg_8_	0.45	0.24	0.06

## Data Availability

Please contact the corresponding author for data related to this article.
